# Role of UBIAD1 in Intracellular Cholesterol Metabolism and Vascular Cell Calcification

**DOI:** 10.1371/journal.pone.0149639

**Published:** 2016-02-18

**Authors:** Sha Liu, Wang Guo, Xue Han, Wendi Dai, Zongli Diao, Wenhu Liu

**Affiliations:** Department of Nephrology, Beijing Friendship Hospital, Faculty of Nephrology, Capital Medical University, Xicheng District, Beijing, China; University of California, Los Angeles, UNITED STATES

## Abstract

Vascular calcification is an important risk factor associated with mortality among patients with chronic kidney disease. Intracellular cholesterol metabolism is involved in the process of vascular cell calcification. In this study, we investigated the role of UbiA prenyltransferase domain containing 1 (UBIAD1) in intracellular cholesterol metabolism and vascular cell calcification, and identified its subcellular location. Primary human umbilical vein smooth muscle cells (HUVSMCs) were incubated with either growth medium (1.4 mmol/L Pi) or calcification medium (CM) (3.0 mmol/L Pi). Under treatment with CM, HUVSMCs were further incubated with exogenous cholesterol, or menaquinone-4, a product of UBIAD1. The plasmid and small interfering RNA were transfected in HUVSMCs to alter the expression of UBIAD1. Matrix calcium quantitation, alkaline phosphatase activity, intracellular cholesterol level and menaquinone-4 level were measured. The expression of several genes involved in cholesterol metabolism were analyzed. Using an anti-UBIAD1 antibody, an endoplasmic reticulum marker and a Golgi marker, the subcellular location of UBIAD1 in HUVSMCs was analyzed. CM increased matrix calcium, alkaline phosphatase activity and intracellular cholesterol level, and reduced UBIAD1 expression and menaquinone-4 level. Addition of cholesterol contributed to increased matrix calcification and alkaline phosphatase activity in a dose-dependent manner. Elevated expression of UBIAD1 or menaquinone-4 in HUVSMCs treated with CM significantly reduced intracellular cholesterol level, matrix calcification and alkaline phosphatase activity, but increased menaquinone-4 level. Elevated expression of UBIAD1 or menaquinone-4 reduced the gene expression of sterol regulatory element-binding protein-2, and increased gene expression of ATP binding cassette transporters A1, which are in charge of cholesterol synthesis and efflux. UBIAD1 co-localized with the endoplasmic reticulum marker and the Golgi marker in HUVSMCs. In conclusion, high intracellular cholesterol content contributes to phosphate-induced vascular cell differentiation and calcification. UBIAD1 or menaquinone-4 could decrease vascular cell differentiation and calcification, probably via its potent role of inversely modulating cellular cholesterol.

## Introduction

Vascular calcification (VC) is prevalent among patients with chronic kidney disease and is associated with increased risk of cardiovascular morbidity and mortality [1–5]. Numerous studies have shown that VC is an active, highly regulated cellular process, mainly mediated by vascular smooth muscle cells (VSMCs) [6,7]. One key event involved in VC promotion is an increase in extracellular calcium and phosphate, which leads to the transition of VSMCs from a contractile to an osteo-chondrogenic phenotype with the formation of a pro-calcifying matrix and vesicles able to nucleate hydroxyapatite. This process is also associated with the loss of mineralization inhibitors, including matrix gla protein and pyrophosphate, calcium- and phosphate-dependent cell death, the production of apoptotic bodies, and the development of mineral nucleation [6,7]. However, the precise mechanisms of VC are still poorly understood.

Cholesterol is an integral component of cell membranes and matrix vesicles. Cells obtain cholesterol by endogenous synthesis or uptake from the extracellular milieu in a tightly regulated homeostatic process. Because cholesterol content is greater in matrix vesicles than in the cytoplasm, vesicle production may drain the cell of its cholesterol stores unless cholesterol synthesis is up-regulated [8]. Up-regulation of cholesterol metabolism plays an essential role in matrix mineralization in VSMCs [8]. A recent study showed that a decreased level of intracellular cholesterol resulted in attenuated matrix mineralization and osteogenic differentiation, which was induced by the protein kinase A agonist in murine aortic cells or spontaneous osteogenic differentiation and mineralization in bovine calcifying vascular cells [8]. In classic high-phosphate induced VC, the role of intracellular cholesterol metabolism remains unknown.

UbiA prenyltransferase domain containing 1 (UBIAD1), also known as transitional epithelial response gene (TERE1), was first identified in 2001 as a tumor suppressor for human bladder carcinoma [9,10]. Later studies showed that the UBIAD1 gene encodes a class of UbiA prenyltransferase involved in Schnyder’s corneal dystrophy, a rare dominant genetic eye disease [11–13]. The main phenotype of Schnyder’s corneal dystrophy is the local accumulation of cholesterol, causing progressive corneal pacification [14,15]. Further studies showed that UBIAD1 protein can modulate intracellular cholesterol and lower the intracellular cholesterol level in HEK293 cells and cancer cells [16–19]. In addition, UBIAD1 was the first enzyme shown to be responsible for human vitamin K biosynthesis [20], converting vitamin K derivatives to menaquinone-4 (MK-4). MK-4, a type of vitamin K_2_, known as a cofactor for γ-carboxyglutamyl carboxylase and a potent activator of steroid and xenobiotic-sensing nuclear receptor (SXR), is involved in multiple biological functions as diverse as bone and cardiovascular mineralization, vascular hemostasis, energy metabolism, immune response, cellular growth, survival and signaling [21].

Although UBIAD1 contributes to several vital functions, the subcellular location of UBIAD1 in different cell types remains controversial [20,22–24], and little is known about the function of UBIAD1 in VSMCs. In this study, we investigated the role of UBIAD1 in intracellular cholesterol metabolism and VSMC calcification and osteogenic differentiation induced by high phosphate, as well as the subcellular location of UBIAD1 in VSMCs.

## Materials and Methods

### Cell culture

Primary human umbilical vein smooth muscle cells (HUVSMCs) were purchased from ScienCell Research Laboratories (Carlsbad, CA, USA). Cells were cultured in smooth muscle cell medium (ScienCell) containing 2% fetal bovine serum. Cells of passages 3–5 were used. HUVSMCs were incubated with either growth medium (GM, 1.4 mmol/L Pi) or calcification medium (CM, 3.0 mmol/L Pi). Media were changed every 2 days. Under treatment with CM, HUVSMCs were further incubated with different concentrations of water-soluble cholesterol, a cholesterol-cyclodextrin complex (Sigma-Aldrich, St. Louis, MO, USA) or exogenous MK-4 (15 μmol /L) (Sigma-Aldrich) for 72 h, to clarify the effect of cholesterol or MK-4 on intracellular cholesterol metabolism and vascular cell calcification.

### siRNA transfection

The small interfering RNA duplex specific to UBIAD1 (siUBIAD1) is a pool of three target-specific 19–25 nt siRNAs designed to knock down gene expression (Santa Cruz Biotechnology Inc., Dallas, TX, USA). For control siRNA (siControl), we used a reference scrambled siRNA (Santa Cruz Biotechnology Inc.). HUVSMCs were plated in 6-well plates and transfected with 6 μl siUBIAD1 or siControl, and 6 μl siRNA transfection reagent (Santa Cruz Biotechnology Inc.) in accordance with the siRNA transfection protocol. Briefly, the cells were first incubated with the siRNA transfection mixture in serum-free medium for 16 h, and then were subsequently cultured with the addition of the normal GM containing 2 times the normal serum. Experimental analyses were performed 48 h after the initiation of transfection.

### Plasmid transfection

The plasmid UBIAD1-EGFP (pUBIAD1) was constructed by subcloning the full-length UBIAD1 cDNA into the enhanced green fluorescent protein (EGFP) vector plasmid (EGFP-N1), as reported previously [22]. The EGFP-N1 plasmid was used as the control (pControl). Both plasmids were kindly provided by Prof. Ling Hong at the Department of Genetics and Developmental Biology, College of Life Science and Technology, Huazhong University of Science and Technology, China. Cells were plated in 6-well plates and transfected with 2 μg plasmid DNA and 6 μl Fugene HD (Promega Corp., Madison, WI, USA) according to the Fugene HD manual. Experimental analyses were performed 48 h after the initiation of transfection.

### Western blotting

Western blot analysis was performed as described previously [9]. The UBIAD1 antibody was a UBIAD1-specific affinity-purified polyclonal antibody raised in rabbits against a UBIAD1-specific peptide (1:500) (Abcam PLC, Cambridge, UK). The peroxidase-conjugated secondary antibody was rabbit immunoglobulin raised in goat (Santa Cruz Biotechnology Inc.). Western blots were processed using an electrochemiluminescent detection system (Nacalai Tesque, Kyoto, Japan).

### Matrix calcium quantitation

Matrix calcium quantitation was analyzed using the o-cresolphthalein complex one method (Teco Diagnostics, Anaheim, CA, USA) and normalized to total protein using the Bradford method [25].

### Alkaline phosphatase activity

Alkaline phosphatase (ALP) activity was used as a biomarker of osteogenic differentiation in VSMCs. ALP activity was assessed colorimetrically using an ALP Activity Assay kit (Nanjing Jiancheng Chemical Industrial Co., Nanjing, China), and normalized to total protein using the Bradford method [25]. Lysates were prepared according to the manufacturer’s instructions.

### Intracellular cholesterol measurement

Total cellular cholesterol content was measured using the Amplex Red cholesterol assay kit (Invitrogen Corp., Carlsbad, CA, USA). Lysates were prepared as described previously [16].

### UPLC-MS/MS analysis

HUVSMCs cultured on 6-well plates were collected and washed three times with cold PBS (Ca^2+^ and Mg^2+^ free) and then stored at -30°C. After being warmed to 20–25°C, cells were lysed in 1 ml of PBS and protein concentrations were determined using the Bradford method [25]. Extraction of MK-4 from cell lysate was performed as reported previously [26] and MK-4 was measured by ultra-performance liquid chromatography–tandem mass spectrometry (UPLC-MS/MS). MK-4 (Sigma-Aldrich) was used as a standard.

Chromatographic separation was performed on a SB-C_18_ column (2.1 mm × 50 mm, 1.8 μm) (Agilent Technologies, Santa Clara, CA,USA) using an Agilent 1290 Series Ultra Performance Liquid Chromatography system (Agilent Technologies) equipped with a binary gradient pump, autosampler, column oven and diode array detector. The column was maintained at 25°C. Isocratic elution was used for the separation, with a mobile acetonitrile (100%) phase and a flow rate of 0.5 mL/min. The injected sample volume was 5 μL, and the analysis took 3 min. Mass spectrometry experiments were performed on an Agilent 6490 UPLC-MS/MS, with an atmospheric pressure chemical ionization source. The measurement conditions were as follows: gas temperature, 200°C; vaporizer 350°C; gas flow, 14 L/min; nebulizer gas, 20 psi; and capillary, 4500 V.

The MS/MS detection was performed using multiple reaction monitoring, in positive mode. The mass transitions of the protonated precursor/product ion pairs that were used to record the selected ion mass chromatograms of MK-4 were *m/z* 445.3 → 187.1 (quantitative ions; collision energy, 20 V) and 445.3 → 227 (qualitative ions; collision energy, 20 V). Data acquisition and processing were performed using MassHunter workstation B.06.00 (Agilent Technologies).

### Alizarin Red S staining

Alizarin Red S staining was used to show the matrix calcification morphologically. HUVSMCs plated in 12-well plates were cultured in GM or CM for 14 d. Briefly, cells were fixed in 95% ethanol for 30 min at room temperature and stained with 40 mmol/L Alizarin Red S for 10 min. Next, cell preparations were washed with PBS to eliminate nonspecific staining. Stained matrix was observed under microscope.

### Quantitative real-time PCR

RNA isolation and quantitative real-time PCR were performed as described previously [27]. Briefly, total RNA was isolated, reverse transcribed, and PCR was performed using SYBR Green PCR Master Mix (Applied Biosystems, Foster City, CA, USA) and the following primers: low density lipoprotein receptor (LDLR) forward 5′- AATGCTTGGACAACAACGGC-3′ and reverse 5′-CGGGATCCTGAC ACTCATC G-3′; HMG-COA reductase (HMGCR) forward 5′- AGCCTGGGCCA GAGAAG ATA-3′ and reverse 5′-GGCACAGTTCTAGGGCCATT-3′; ATP binding cassette transporters A1 (ABCA1) forward 5′- GATGGCAATCATGGTCAATGG -3′ and reverse 5′- AGCTGGTATTGTAGCATGTTCCG -3′; and sterol regulatory element-binding protein-2 (SREBP-2) forward 5′- GATGCGGAGAAGCTGCCTAT -3′ and reverse 5′- GCTGTG TTGCAGAAAGCGAA -3′.

### Immunofluorescence

To observe endogenous UBIAD1 expression in HUVSMCs, cells were mounted onto polylysine slides, treated with formaldehyde and blocked. Slides were incubated with anti-UBIAD1 antibody (1:50) (Abcam PLC) overnight at 4°C. Following several washes with PBS, the slides were incubated with green FITC conjugated secondary antibody (goat anti-rabbit IgG (H+L)) (1:100) (Protein Tech Group Inc., Chicago, IL, USA) at 37°C for 60 min. Fluorescent expression of UBIAD1 in HUVSMCs was observed by confocal microscopy.

The subcellular location of UBIAD1 was further analyzed. HUVSMCs mounted onto polylysine-slides were stained with the endoplasmic reticulum (ER) marker, ER-tracker Red or the Golgi marker, BODIPY-TR ceramide (Thermo Fisher Scientific, Waltham, MA, USA), and then treated with formaldehyde. Slides were blocked and incubated with anti-UBIAD1 antibody and green FITC conjugated secondary antibody subsequently, followed by staining of nuclei with 4′, 6-diamidino-2-phenylindole (DAPI) (Thermo Fisher Scientific). The merged fluorescent images were analyzed by confocal microscopy to identify the co-localization of UBIAD1 with intracellular organelles.

### Statistical analysis

Each experiment was performed in at least quadruplicate wells and repeated at least three times. Two-way analysis of variance was used to analyze the effects of two factors on the results, e.g. effects of time and media on markers, or the effects of plasmids and cholesterol on markers. Student’s t test was used for comparisons between two groups. Pearson correlation analysis was used to identify the linear relationship between two variables. A value of *P* < 0.05 was considered significant.

## Results

### Effects of CM on matrix calcification, osteoblastic differentiation, intracellular cholesterol, UBIAD1 expression and MK-4 level

**Fig 1:**
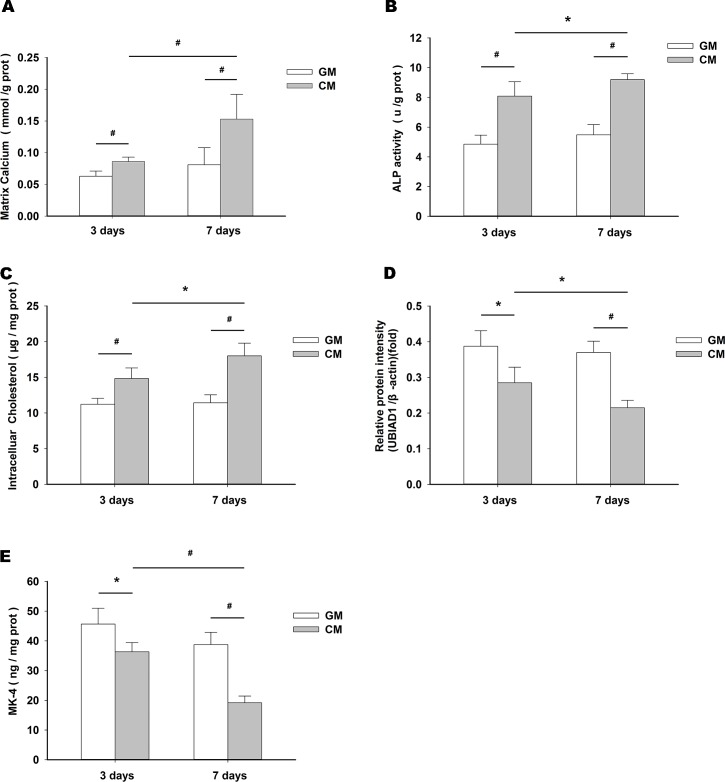
Effects of CM on matrix calcification, osteoblastic differentiation, intracellular cholesterol, UBIAD1 expression and MK-4 level. Increased matrix calcium (A), ALP activity (B), and intracellular cholesterol (C) levels, and reduced UBIAD1 expression (D), and MK-4 level (E) in HUVSMCs treated with CM for 3 d and 7 d, compared with levels in cells treated with GM.*P < 0.05, ^#^*P* < 0.01.

Treatment of HUVSMCs with CM for 3–7 d induced matrix calcification (Fig 1A) and ALP activity (Fig 1B), compared with treatment using GM. HUVSMCs treated with CM also showed increased intracellular cholesterol level (Fig 1C). We also observed that CM treatment reduced UBIAD1 expression (Fig 1D) and MK-4 level (Fig 1E).

**Fig 2:**
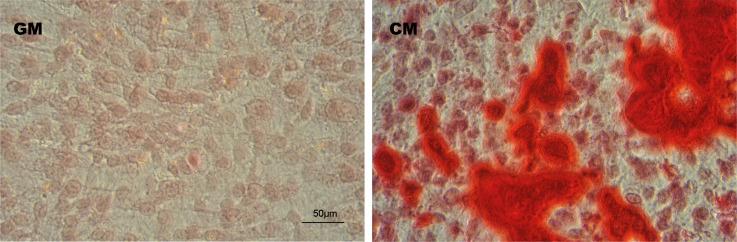
Effects of CM on matrix calcification identified by Alizarin Red S staining. Images of the Alizarin Red S staining to show the mineral deposition in matrix of HUVSMCs cultured in GM or CM for 14 d.

Matrix calcification were further identified by Alizarin Red S staining. Upon treatment of HUVSMCs with CM for 14 d, abundant mineral deposits could be detected in the extracellular matrix. In comparison, much less mineral deposition was observed in HUVSMCs treated with GM (Fig 2).

### Effects of cholesterol uptake on phosphorus-induced matrix calcification, ALP activity and intracellular cholesterol

**Fig 3:**
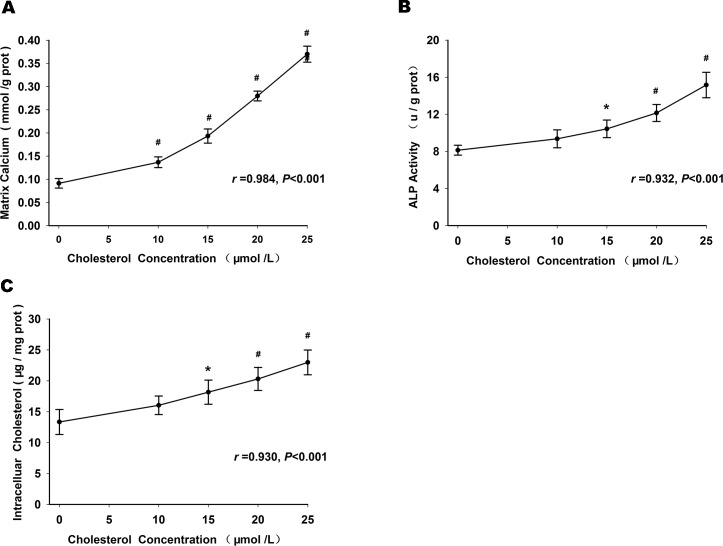
Effects of cholesterol concentration on matrix calcification and osteoblastic differentiation. Matrix calcium (A), ALP activity (B) and intracellular cholesterol (C) increased in a dose-dependent manner in HUVSMCs treated with CM with the addition of cell-permeable cholesterol for 72 h. *P < 0.05, ^#^*P* < 0.01, compared with cholesterol 0 μmol/L.

We also tested the effects of cholesterol uptake on phosphorus-induced matrix calcification, ALP activity and intracellular cholesterol by culturing HUVSMCs under CM with concentrations of cholesterol ranging from 0–25 μmol/L. Phosphorus-induced matrix calcium (Fig 3A), ALP activity (Fig 3B) and intracellular cholesterol (Fig 3C) increased in a dose-dependent manner in HUVSMCs treated with CM with the addition of cell-permeable cholesterol for 72 h. Treatment with cyclodextrin alone did not have any significant effect (data not shown).

### Decreased expression of UBIAD1 increases matrix calcification, osteoblastic differentiation, and intracellular cholesterol and reduces MK-4 level in HUVSMCs

**Fig 4:**
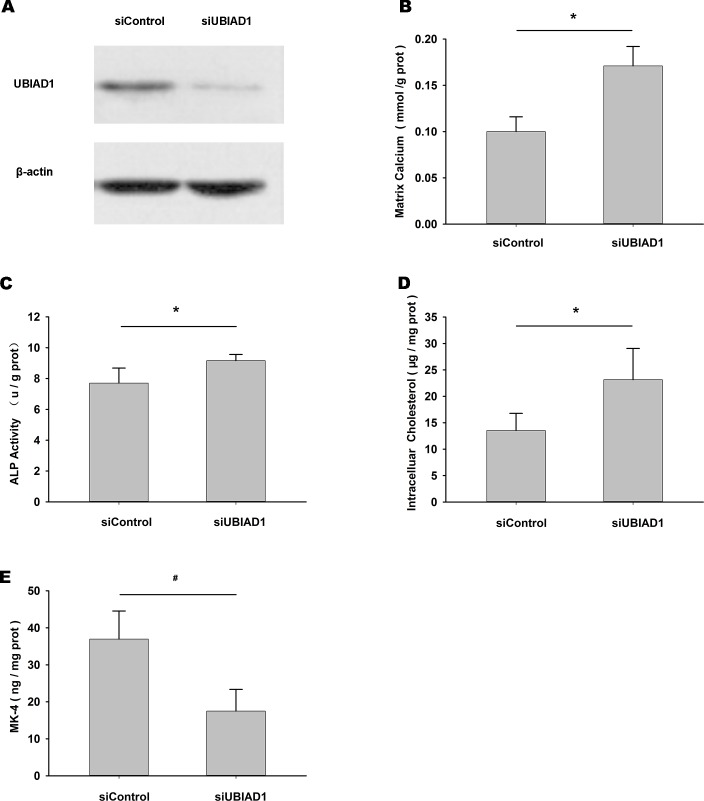
Decreased expression of UBIAD1 increased matrix calcification, osteoblastic differentiation, and intracellular cholesterol levels and reduced MK-4 level. (A) Endogenous levels of UBIAD1 and β-actin after transfection with siControl or siUBIAD1. (B–E) Differences in matrix calcium (B), ALP activity (C), intracellular cholesterol (D) and MK-4 levels (E) after transfection with siControl or siUBIAD1 in HUVSMCs treated with CM for 72 h. *P < 0.05, ^#^*P* < 0.01.

siUBIAD1 was used to reduce the endogenous level of UBIAD1 in HUVSMCs (Fig 4A). Transfection with siUBIAD1 caused a significant increase in matrix calcium (Fig 4B), ALP activity (Fig 4C) and intracellular cholesterol (Fig 4D) level and reduced MK-4 level (Fig 4E) in HUVSMCs treated with CM for 72 h, compared with siControl.

### Elevated expression of UBIAD1 or exogenous MK-4 reduces matrix calcification, osteoblastic differentiation, and intracellular cholesterol, and increases MK-4 level in HUVSMCs

**Fig 5:**
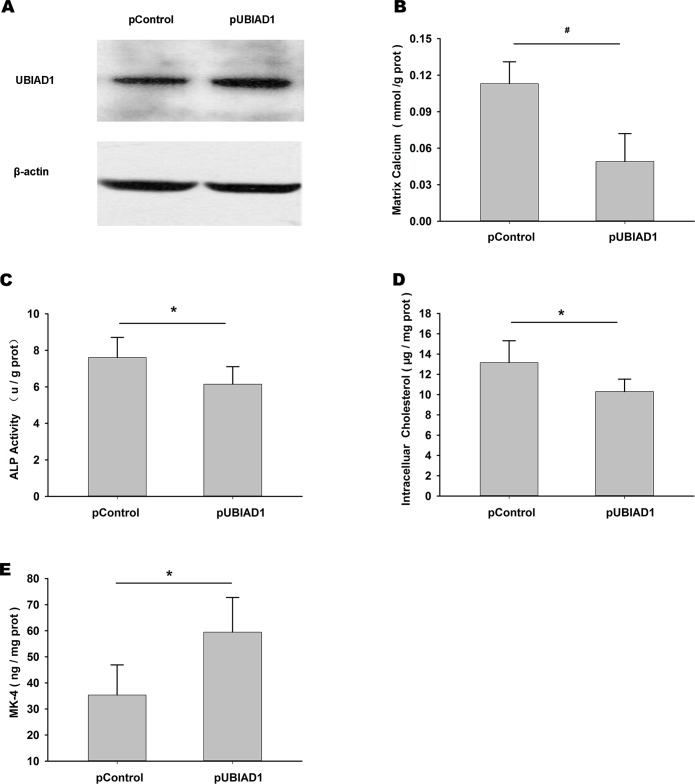
Elevated expression of UBIAD1 reduced matrix calcification, ALP activity and intracellular cholesterol levels, and increased MK-4 level. (A) Endogenous levels of UBIAD1 and β-actin after transfection with pControl or pUBIAD1. (B–E) Differences in matrix calcium (B), ALP activity (C), intracellular cholesterol (D) and MK-4 level (E) after transfection with pControl or pUBIAD1 in HUVSMCs treated with CM for 72 h. *P < 0.05, ^#^*P* < 0.01.

After transfection of pUBIAD1, we observed increased expression of UBIAD1 in HUVSMCs (Fig 5A). Transfection with pUBIAD1 caused significantly reduced matrix calcium (Fig 5B), ALP activity (Fig 5C) and intracellular cholesterol (Fig 5D), and increased MK-4 levels (Fig 5E) in HUVSMCs treated with CM for 72 h, compared with the pControl.

**Fig 6:**
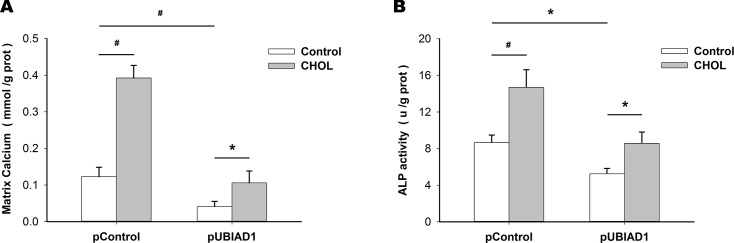
Effects of pUBIAD1 on matrix calcification and osteoblastic differentiation were attenuated by adding cell-permeable cholesterol. Matrix calcium (A) and ALP activity (B) in HUVSMCs after transfection with pControl or pUBIAD1, with the addition of blank control or cholesterol (CHOL) (25 μmol/L) in HUVSMCs treated with CM for 72 h. *P < 0.05, ^#^*P* < 0.01.

However, the effects of pUBIAD1 on matrix calcium (Fig 6A) and ALP activity (Fig 6B) in HUVSMCs treated with CM were attenuated by adding cell-permeable cholesterol (25 μmol/L) for 72 h.

**Fig 7:**
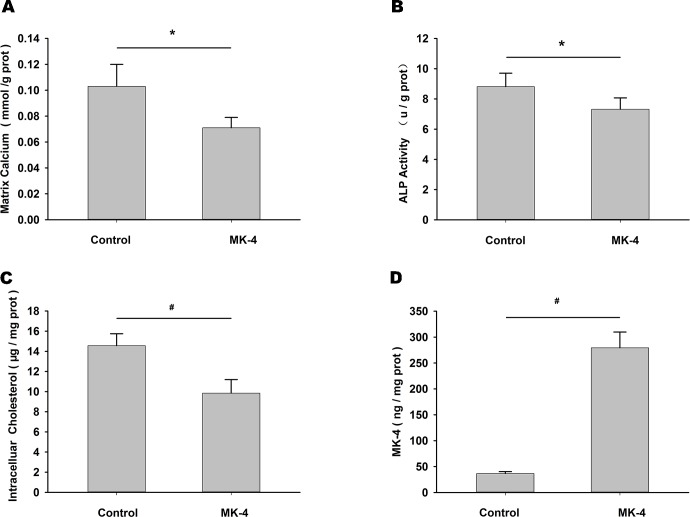
Exogenous MK-4 reduced intracellular cholesterol levels, matrix calcification and osteoblastic differentiation. Differences in matrix calcium (A), ALP activity (B), intracellular cholesterol (C) and MK-4 level (D) after addition of blank control or exogenous MK-4 (15 μmol /L) in HUVSMCs treated with CM for 72 h. *P < 0.05, ^#^*P* < 0.01.

Similarly, in CM-treated HUVSMCs, addition of exogenous MK-4 (15 μmol /L) for 72 h reduced matrix calcium (Fig 7A), ALP activity (Fig 7B) and intracellular cholesterol levels (Fig 7C), and increased MK-4 level (Fig 7D) compared with the blank control.

### UBIAD1 or MK-4 induced changes in the expression of genes involved in cholesterol metabolism

**Fig 8:**
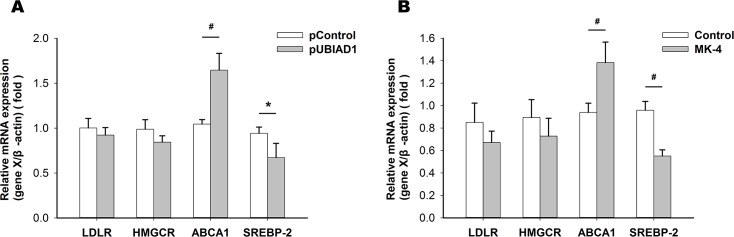
UBIAD1 or MK-4 induced changes in the expression of genes involved in cholesterol metabolism. Changes in the expression of LDLR, HMGCR, ABCA1 and SREBP-2 genes after transfection with pUBIAD1 (A) or addition of MK-4 (15 μmol /L) (B) in HUVSMCs treated with CM for 72 h. *P < 0.05, ^#^*P* < 0.01.

We next explored the underlying causes of the decreased intracellular cholesterol level in HUVSMCs treated with pUBIAD1 or MK-4. We analyzed the expression of several genes involved in cholesterol metabolism using quantitative real-time PCR. We found that the mRNA expression of SREBP-2, a gene involved in cholesterol synthesis, was reduced after transfection with pUBIAD1 (Fig 8A) or addition of MK-4 (15 μmol /L) (Fig 8B) in HUVSMCs treated with CM for 72 h, whereas mRNA expression of ABCA1, a gene involved in cholesterol efflux, was elevated compared with the control. There were no significant changes of the mRNA expression of LDLR and HMGCR genes (Fig 8).

### UBIAD1 is localized to the ER and Golgi

**Fig 9:**
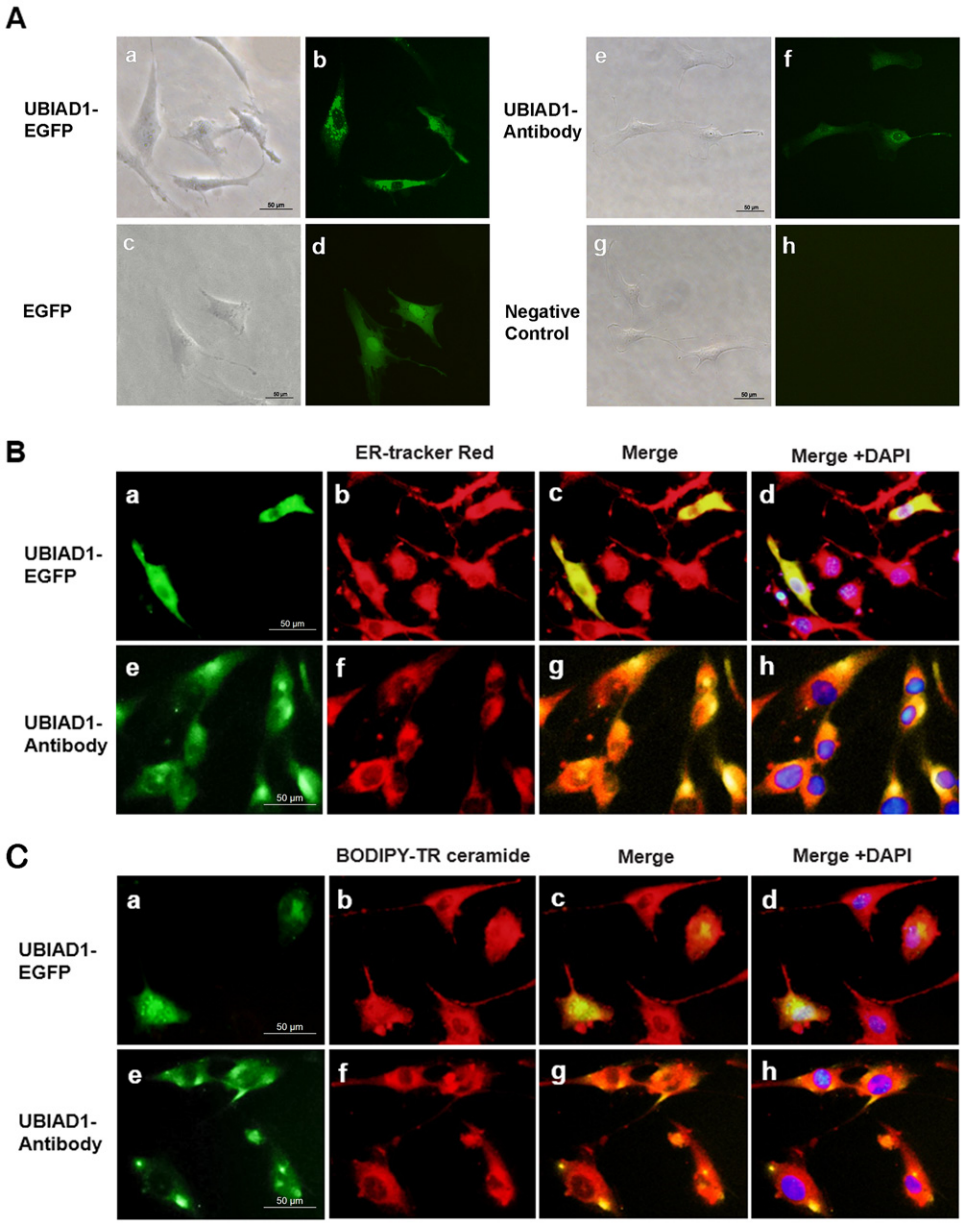
UBIAD1 localizes to the endoplasmic reticulum (ER) and the Golgi in HUVSMCs. (A) UBIAD1 expressed in the cytoplasm of HUVSMCs: (a–b) HUVSMCs transfected with UBIAD1-EGFP plasmid; (c–d) HUVSMCs transfected with EGFP plasmid as control; (e–f) HUVSMCs stained with UBIAD1 antibody and the FITC-conjugated secondary antibody; and (g–h) HUVSMCs stained with the absent UBIAD1 antibody as the negative control. (B) UBIAD1 localized to the ER in HUVSMCs: (a) UBIAD1-EGFP expressed in HUVSMCs; (b and f) the ER marker, ER-tracker red; (c) merged image of a and b; (d) image c with nuclear DAPI staining; (e) HUVSMCs stained with UBIAD1 antibody and the FITC-conjugated secondary antibody; (g) merged image of e and f; (h) image g with nuclear DAPI staining. (C) UBIAD1 localized to the Golgi in HUVSMCs: (a) UBIAD1-EGFP expressed in HUVSMCs; (b and f) the Golgi marker, BODIPY-TR ceramide; (c) merged image of a and b; (d) image c with nuclear DAPI staining; (e) HUVSMCs stained with UBIAD1 antibody and the FITC-conjugated secondary antibody; (g) merged image of e and f; (h) image g with nuclear DAPI staining.

We next examined the subcellular localization of UBIAD1 in HUVSMCs. Transfection of pUBIAD1 in HUVSMCs revealed an abundance of UBIAD1 in a mesh-like structure of the cytoplasm (Fig 9A a–b), whereas the fluorescence in cells transfected with pControl presented with a more evenly diffuse pattern throughout the cytoplasm and nuclear (Fig 9A c–d). Ectopically expressed UBIAD1 co-localized with the ER marker (ER-tracker Red) (Fig 9B a–d) and Golgi marker (BODIPY-TR ceramide) (Fig 9C a–d) outside the nucleus. These results strongly indicate that exogenous UBIAD1 is localized to both the ER and Golgi.

To confirm that the ER and Golgi localization of UBIAD1 in HUVSMCs was not an artifact of protein overexpression, we used the UBIAD1 antibody to study subcellular localization of endogenous UBIAD1. Patchy aggregations of endogenous UBIAD1 were observed inside HUVSMCs (Fig 9A e–f), compared with the negative control (Fig 9A g–h). UBIAD1 also co-localized with the ER marker (Fig 9B e–h) and Golgi marker (Fig 9C e–h) outside the nucleus. These data confirm that endogenous UBIAD1 is localized to the ER and Golgi. This matches the pUBIAD1 expression pattern and further supports localization of UBIAD1 in the ER and Golgi.

## Discussion

In this study, we investigated the role of UBIAD1 in intracellular cholesterol metabolism, vascular cell calcification and osteogenic differentiation, as well as the subcellular location of UBIAD1 in VSMCs. High phosphate concentration has been shown to induce VC and phenotypic conversion of VSMCs [7,28–30]. In response to high phosphate stimulus, VSMCs show decreased expression of VSMC differentiation markers and increased expression of osteogenic markers. Our results confirm that high phosphate can induce increased ALP activity and matrix calcification, and are consistent with previous studies [7,28–30]. Furthermore, we revealed the role of cholesterol metabolism in vascular cell calcification induced by high phosphate. During the processes of high phosphate-induced VSMC differentiation and calcification, intracellular cholesterol content increased. High intracellular cholesterol level was positively correlated with a higher level of phosphate-induced calcification. Intracellular cholesterol is incorporated into matrix vesicles, which are exported as part of the process of extracellular matrix mineralization. Inhibition of intracellular cholesterol metabolism could be a potential strategy to attenuate mineralization.

UBIAD1 is a potent intracellular cholesterol metabolism suppressor. In HEK293 cells and bladder, prostate and renal cancer cells, elevated UBIAD1 expression reduced cholesterol levels [16, 28–30]. In the present study, UBIAD1 plasmid and siRNA specific to UBIAD1 were introduced to alter the protein expression in HUVSMCs. Increased UBIAD1 expression or addition of its product MK-4 not only reduced cholesterol levels, which is in accordance with previous work [16,28–30], but also reduced ALP activity and matrix calcium levels under CM treatment. In contrast, transfection with siUBIAD1 caused a significant increase in matrix calcium, ALP activity, and intracellular cholesterol level. Together with the findings of the decreased expression of UBIAD1 and MK-4 level in the processes of high phosphate-induced VSMC differentiation and mineralization, our study confirmed that UBIAD1 plays an important role in cholesterol metabolism and vascular cell calcification. The effect of UBIAD1 on cholesterol metabolism, ALP activity and matrix calcification, was partially attenuated by the addition of water-soluble cholesterol in VSMCs, which reiterates the role of cholesterol in phosphorus-induced VSMC calcification. Above all, our results suggest that UBIAD1 attenuates mineralization of VSMCs possibly via inhibition of intracellular cholesterol metabolism.

Cellular cholesterol levels are generally highly regulated by several processes: transport (influx and efflux), de novo synthesis, trafficking, storage, recycling and catabolism [31–33]. Exogenous cholesterol enters the cell via LDLR, and is transported to the ER for processing. In the ER, HMGCR catalyzes a rate-limiting step of de novo synthesis. The major energy dependent mechanism for cholesterol efflux is via ATP binding cassette transporters on the cell surface. ABCA1 is a major cholesterol efflux regulatory protein, which can deliver cholesterol to apolipoproteins AI and apolipoproteins E. Excess intracellular cholesterol is stored as unesterified cholesterol or cholesterol ester, and is regulated by the ER enzyme sterol O-acyltransferase (SOAT1). Within the ER also resides SREBP, which interacts with SREBP cleavage activating protein (Scap) in the ER. The Scap/SREBP complex is then transported to the Golgi apparatus via CopII proteins [34]. Once in the Golgi, SREBP undergoes proteolytic cleavage and the N-terminus is released, acting as a nuclear transcription factor, with a consequential increase in cholesterol production [33].

The mechanism by which UBIAD1 modulates cholesterol homeostasis is not yet well defined. UBIAD1 may directly interact with HMGCR, SOAT1, and apolipoproteins E, which are mediators of synthesis, storage, and efflux [35, 36], and act as the elusive target of geranylgeraniol in ER-associated degradation of HMGCR [37]. MK-4 is a potent activator of SXR. By regulating the endogenous levels of MK-4, UBIAD1 controls the transcription of a suite of SXR target genes, such as ABCA1, HMGCR and sterol 27-hydroxylase, that govern cholesterol efflux and steroid catabolism [17]. As indicated by our results, both the decreased synthesis (SREBP-2), and increased efflux (ABCA1) likely account for the decreased intracellular cholesterol induced by pUBIAD1 we observed, which are consistent with previous work[17–19]. Considering the possible mediating role of MK-4 in the UBIAD1-regulating cholesterol mechanisms, similar results were observed when MK-4 was included alone, reinforcing that a MK-4/SXR mechanism is in effect. However, unlike the increased expression of the HMGCR gene induced by UBIAD1 or MK-4 reported by others [18,19], we did not observe any significant changes in the expression of the HMGCR gene. These conflicting results regarding the expression of the HMGCR gene and reduced intracellular cholesterol would suggest a more complicated relationship between UBIAD1 and HMGCR besides the classical MK-4/SXR mechanism, such as UBIAD1- mediated ER-associated degradation of HMGCR [37].

Several groups have independently reported the subcellular localization of UBIAD1 in different cells [20,22–24]. Subcellular localization of protein determines its biological function. Nakagawa et al. showed that UBIAD1, acting as a biosynthetic enzyme for MK-4, is localized to the ER rather than the Golgi in human osteoblast-like MG-63 cells [20]. Nickerson et al. showed that UBIAD1 is localized to mitochondria but not to the ER in human keratocytes [24]. Wang et al. found that UBIAD1 accumulates on the Golgi in human bladder carcinoma cells and its localization partially influenced the tumor suppressing activity [22]. Mugoni et al. showed that UBIAD1 is localized to the Golgi membranes, responsible for CoQ10 production, but not to mitochondria in human endothelial cells [23]. Our results show that both exogenous and endogenous UBIAD1 localize to the ER and Golgi in HUVSMCs. The subcellular localization of UBIAD1 in HUVSMCs is in accordance with its biological function in cholesterol metabolism. The ER and Golgi are the two most important intracellular organelles involved in the homeostasis of intracellular cholesterol. In the ER, UBIAD1 converts vitamin K derivatives to MK-4 [20], a mediator that affects cholesterol synthesis, efflux and catabolism via SXR. In the ER and Golgi, UBIAD1 may interact with HMGCR and SOAT1, and thus affect cholesterol synthesis and storage [35].A previous report showed that wild-type UBIAD1 was transported via the endomembrane system from the ER to the Golgi in bladder carcinoma cells, while mutant proteins could not be transported [22]. The role of translocation in regulating cholesterol metabolism is still under investigation.

In conclusion, we observed increased intracellular cholesterol, and decreased UBIAD1 expression and MK-4 level in the processes of high phosphate-induced VSMC differentiation and mineralization. High intracellular cholesterol content contributed to phosphate-induced vascular cell differentiation and calcification. UBIAD1 or MK-4 could decrease vascular cell differentiation and calcification, probably via its potent role of inversely modulating cellular cholesterol. The decreased intracellular cholesterol induced by UBIAD1 or MK-4 would likely be a result of the decreased synthesis, and increased efflux. UBIAD1 is localized to the ER and Golgi in HUVSMCs. These findings reveal the role of UBIAD1 in intracellular cholesterol metabolism and VC and provide a possible novel treatment target. The exact molecular mechanism and prolonged effects of UBIAD1 on VC require further exploration.
